# Assessment of dual tasking has no clinical value for fall prediction in Parkinson’s disease

**DOI:** 10.1007/s00415-012-6419-4

**Published:** 2012-02-01

**Authors:** Katrijn Smulders, Rianne A. J. Esselink, Aner Weiss, Roy P. C. Kessels, Alexander C. H. Geurts, Bastiaan R. Bloem

**Affiliations:** 1 Department of Neurology, Radboud University Nijmegen Medical Centre, Donders Institute for Brain, Cognition and Behaviour, Internal code 935, P.O. Box 9101, 6500 HB Nijmegen, The Netherlands; 2 HAN University of Applied Sciences, Institute for Studies in Sports and Exercise, Nijmegen, The Netherland; 3 Movement Disorders Unit, Department of Neurology, Tel-Aviv Sourasky Medical Centre, Tel-Aviv, Israel; 4 Department of Medical Psychology, Radboud University Nijmegen Medical Centre, Nijmegen, The Netherland; 5 Radboud University Nijmegen, Donders Institute for Brain, Cognition and Behaviour, Nijmegen, The Netherland; 6 Department of Rehabilitation, Radboud University Nijmegen Medical Centre, Nijmegen Centre for Evidence Based Practice, Nijmegen, The Netherlands; 7 Sint Maartenskliniek, Research, Development and Education, Nijmegen, The Netherlands

**Keywords:** Dual task, Falls, Gait, Parkinson’s disease, Executive function

## Abstract

The objective of this study is to investigate the value of dual-task performance for the prediction of falls in patients with Parkinson’s disease (PD). Two hundred sixty-three patients with PD (H&Y 1–3, 65.2 ± 7.9 years) walked two times along a 10-m trajectory, both under single-task and dual-task (DT) conditions (combined with an auditory Stroop task). To control for a cueing effect, Stroop stimuli were presented at variable or fixed 1- or 2-s intervals. The auditory Stroop task was also performed alone. Dual-task costs were calculated for gait speed, stride length, stride time, stride time variability, step and stride regularity, step symmetry and Stroop composite scores (accuracy/reaction time). Subsequently, falls were registered prospectively for 1 year (monthly assessments). Patients were categorized as non-recurrent fallers (no or 1 fall) or recurrent fallers (>1 falls). Recurrent fallers (35%) had a significantly higher disease severity, lower MMSE scores, and higher Timed “Up & Go” test scores than non-recurrent fallers. Under DT conditions, gait speed and stride lengths were significantly decreased. Stride time, stride time variability, step and stride regularity, and step symmetry did not change under DT conditions. Stroop dual-task costs were only significant for the 2-s Stroop interval trials. Importantly, recurrent fallers did not show different dual-task costs compared to non-recurrent fallers on any of the gait or Stroop parameters. These results did not change after correction for baseline group differences. Deterioration of gait or Stroop performance under dual-task conditions was not associated with prospective falls in this large sample of patients with PD.

## Introduction

Falling is a common and incapacitating complication of Parkinson’s disease (PD) [1]. Even in early disease stages a considerable number of patients with PD fall [2]. To identify these fallers, it is necessary to develop a sensitive and specific measure to predict which patients are at high risk of future falls in a timely manner. This is still not adequately possible using existing prediction algorithms.

Lundin-Olsson was the first to demonstrate that older people who stop walking while talking had a higher risk of falling than those who are able to continue walking [3]. Since then, the dual-task paradigm has been regarded as a promising way to discriminate between people at risk of falls and those who are not [4]. Gait deficits generally call for increased attentional demands in order to maintain stability and prevent stumbling. A well-proven paradigm to assess attentional demands of gait is to add a secondary cognitive task and to compute the cost of dual tasking [5, 6]. That is, performing a cognitive task while walking leads to a situation in which two tasks compete for the same attentional resources [7]. When the attentional demands of both tasks together exceed the available capacity, the performance of one or both tasks will deteriorate compared to the respective single-task performance.

In patients with PD, dual-task situations are thought to be especially challenging since executive function is often impaired even in early stages of the disease [8]. Specifically, PD affects the ability to flexibly switch from one attentional set to another [9, 10]. Impaired set-shifting further complicates dual-task situations in which attention needs to be properly allocated to the tasks at hand. When people are walking and are concurrently engaged in a cognitive task, the most sensible strategy to maintain stability is to prioritize posture, thereby decreasing the risk of falling. This notion is called the ‘posture first hypothesis’ [5]. However, Bloem and colleagues found that patients with PD actually gave less priority to motor tasks than healthy participants, possibly placing them at a higher risk of falls [11].

In healthy people, gait adaptations under dual-task conditions include slowing of gait speed and reducing stride length [12]. The same adaptations have been observed in patients with PD [13–15], but their gait variability is also increased under dual-task conditions [16, 17]. Furthermore, gait variability in a single-task condition has been associated with fall risks in PD [18]. Taken together, this has led to the suggestion that increased gait variability under dual-task conditions may be a predictor of falling in patients with PD [6, 19].

The aim of the present study was to investigate whether dual-task performance predicts falling in patients with PD. For this purpose, we evaluated a gait task and a cognitive task (auditory Stroop task) during single-task and dual-task conditions in a large cohort of patients that was prospectively monitored for fall incidence. Fall incidence was accurately monitored for a period of 1 year after the functional assessments.

## Methods

### Participants

The sample was a subset of the 586 idiopathic patients with PD participating in the ParkFit study, a multicentre randomized clinical trial aiming to evaluate the effectiveness of a behavioral program promoting physical activity [20]. Eligibility criteria of the ParkFit study were idiopathic PD with Hoehn and Yahr ≤3, aged between 40 and 75 years with a sedentary lifestyle. Exclusion criteria were: unclear diagnosis, MMSE <24, unable to complete Dutch questionnaires, severe co-morbidity, daily institutionalized care, and deep brain surgery. The study was approved by the regional medical ethics committee (CMO region Arnhem-Nijmegen), and patients gave their written informed consent before the first assessment.

Three hundred thirty-two patients participated in the dual-task study. Data were missing for 69 patients because of errors during recording or storing of the Stroop task (*n* = 17) or gait task (*n* = 11), because of weakness of the recorded Stroop response signals (*n* = 6), inability to understand the Stroop task while seated (*n* = 21), or incomplete fall records (*n* = 14). Therefore, analyses were performed on 263 patients (64.6% men; 65.2 ± 7.9 years; Table 1).

**Table 1:** Demographic and clinical characteristics of the participants

	Total (*n* = 263)	Non-recurrent fallers (*n* = 171)	Recurrent fallers (*n* = 91)	*p* value
Age (years)	65.2 ± 7.9	64.6 ± 8.1	66.3 ± 7.5	0.099
Gender (% men)	64.6%	65.7%	62.6%	0.621
UPDRS-III	34.1 ± 9.4	32.7 ± 9.1	36.7 ± 9.4	0.001
H&Y stage (mode)				
1	9 (3%)	6 (4%)	3 (4%)	0.724
2	248 (94%)	163 (95%)	85 (93%)	
3	6 (2%)	3 (2%)	3 (3%)	
MMSE	28.2 ± 1.6	28.3 ± 1.5	27.8 ± 1.7	0.012
Educational level (mode)	3	3	3	0.873
Timed “Up and Go” (s)	9.5 ± 2.9	9.1 ± 2.9	10.3 ± 2.7	0.003
Falls (*n*)	689	48	641	<0.001

*UPDRS-III* Unified Parkinson’s disease rating scale motor examination, *H&Y* Hoehn & Yahr, *MMSE* Mini-mental state examination

### Clinical assessment (Table 1)

To assess the severity of motor symptoms we used the motor section of the Unified Parkinson’s Disease Rating Scale (UPDRS-III) [21]. Hoehn and Yahr [22] staging (H&Y) was used to assess disease stage. A global index of cognitive function was obtained using the Mini-Mental State Examination (MMSE) [23]. Level of education was assessed using six categories, ranging from ‘no education’ (1) to ‘university degree’ (6). The Timed “Up & Go” (TUG) test was used as an index of mobility [24]. In the TUG test the patient has to stand up from a chair, walk 3 m at comfortable speed, turn 180°, walk back to the chair and sit down again as fast as possible while time is recorded.

### Gait task

Subjects were assessed while walking along a regular walkway of 10 m length. Under both single-task and the various dual-task conditions, each subject completed two trials. Subjects were instructed to walk at their normal pace. Gait parameters were measured with a triaxial accelerometer sampling with 100 Hz (Dynaport, McRoberts) attached to the lower back at the pelvis. The Dynaport accelerometer detects steps with 5.6% error and step duration with 9.9% error in patients with PD [25].

Analysis of gait parameters was performed in Matlab (MathWorks). Temporal gait parameters were calculated using heel strike detection algorithms. Gait speed, stride length, stride time, and stride time variability were calculated. Step and stride regularity and step symmetry were derived from frequency analysis of vertical acceleration signals using unbiased autocorrelation [26]. Perfect regularity (i.e., no variability) and symmetry result in correlation coefficients of 1. For all gait parameters, scores over the first and second 10-m walk were averaged.

### Cognitive task

We selected an auditory Stroop task as the secondary cognitive task [27]. During this task participants hear the word “high” or “low” in a high or low pitch, and are instructed to name the pitch of the stimulus, thus ignoring the meaning of the word. Two conditions are defined: congruent stimuli in which the word and pitch are equal (e.g. “high” at a high pitch), and incongruent stimuli in which the two differ (e.g., “high” at a low pitch). Participants were instructed to respond as accurately and as fast as possible. Before actual measurements, a series of ten Stroop stimuli was practiced.

The stimuli were played by a digital recorder (Micro BR, Boss Corp.) and presented through a headphone with an integrated microphone in a mouthpiece (Sennheiser PC130, Sennheiser). The verbal responses of the subjects were recorded and saved on a digital card (sample frequency 44.1 kHz).

Stroop stimuli of three different complexity levels were presented by varying the interval between stimuli: 1-s intervals, 2-s intervals, and variable (1, 2, or 3 s) intervals. The latter condition was introduced to evaluate a possible cueing effect of the Stroop task on gait [28].

The accuracy of all Stroop responses was scored manually. Onsets of verbal responses were detected and visually inspected in Matlab. Verbal reaction time was calculated as the difference between the start of the stimulus and the start of the response. To account for possible speed-accuracy trade-off, a composite score was calculated by dividing accuracy (% correct responses) by verbal reaction time (ms) [29]. Only reaction times of correct answers were used in the composite score.

### Procedure

All subjects performed both the Stroop task and the gait task as a single task and during dual-task conditions. The three single-task conditions of the Stroop task (1 s, 2 s, and variable interval) were tested while patients were seated. During the dual-task conditions, participants walked while simultaneously responding to each of the three Stroop conditions. No instruction with regard to task priority was given.

Half of the participants started with the single-task Stroop and single-task walk followed by the dual-task condition, whereas others started with the dual-task conditions followed by the single-task conditions. The order of the Stroop conditions was counterbalanced between subgroups of patients, but was equal for the single- and dual-task conditions.

### Falls assessment

In the year following the functional assessments, falls were registered monthly using an automated system to monitor falls over the telephone (Falls Telephone, ASK Community Systems). The Falls Telephone called participants every month and asked them how many times they had fallen in the previous month. The Falls Telephone has been tested and found to be a reliable instrument to monitor falls in PD with a sensitivity of 100% and specificity of 78% [30]. To further increase specificity, all fall entries were verified by a personal telephone call of trained research assistants.

Participants were divided into two groups based on the number of falls: patients with no or a single fall over 12 months (non-recurrent fallers) and patients who had fallen more than once during 12 months (recurrent fallers) [31].

### Data analysis

Differences between recurrent and non-recurrent fallers on demographic and clinical characteristics, single-task walking, and single-task Stroop performance were evaluated using Student’s *t* tests for independent samples in the case of continuous variables and χ^2^ tests in the case of categorical variables. In order to remove skewness, single-task and dual-task scores were log-transformed before analysis. Dual-task effects were assessed by a one-sample *t* test.

Dual-task costs for the gait parameters and for the Stroop composite scores were calculated as the ratio between DT and ST performance. Dual-task costs were calculated separately for the three dual-task conditions. Differences in dual-task costs between recurrent and non-recurrent fallers were analyzed with 3 × 2 (Stroop condition × group) ANOVA with repeated measures (ANOVA-RM). In the case of significant main effects, Bonferroni-corrected post hoc analyses were carried out. To correct for baseline differences between groups, ANCOVA-RM analyses were performed with all clinical and demographic variables that were significantly different between groups as co-variates. For all analyses, significance was accepted at *p* < 0.05 (two-sided).

Finally, in order to gain insight into the strategy used under dual-task conditions for both groups, dual-task costs for the Stroop task (2-s interval) were plotted against dual-task costs for walking (gait speed) for each patient. In this plot patients using a posture-first strategy (high cognitive dual-task costs, low motor dual-task costs) are positioned differently compared to patients with a posture-second strategy (equally high dual-task costs for both tasks, or high costs for walking).

## Results

### Baseline characteristics of recurrent fallers versus non-recurrent fallers

One hundred seventy-one patients with PD (65%) appeared to be non-recurrent fallers. The remaining 91 patients (35%) experienced a total of 661 falls. Recurrent fallers had significantly higher UPDRS-III scores (*p* < 0.001), lower MMSE scores (*p* = 0.012), and lower TUG scores (*p* = 0.003). Age (*p* = 0.099), gender (*p* = 0.621), H&Y stage (*p* = 0.724), and educational level (*p* = 0.873) were not significantly different between the groups. Detailed characteristics of the two groups are presented in Table 1.

Gait and Stroop outcome measures of the single-task conditions are presented in Table 2. Recurrent fallers had significantly lower gait speed (*p* = 0.041) and smaller stride length (*p* = 0.012) compared to non-recurrent fallers. Stride time, stride time variability, step and stride regularity, and step symmetry did not differ significantly between groups (all *p* > 0.05). In addition, no (significant) differences between groups were observed for Stroop composite scores on congruent or incongruent stimuli (all *p* ≥ 0.472).

**Table 2:** Single-task gait outcomes and Stroop composite scores

	Non-recurrent fallers	Recurrent fallers	% difference (CI)	*p* value
Gait				
Speed (m s^−1^)	1.00 (0.17)	0.95 (0.17)	5.3 (0.2–10.6)	**0.041**
Stride length (m)	1.26 (0.21)	1.19 (0.20)	5.8 (1.2–10.6)	**0.012**
Stride time (s)	1.13 (0.11)	1.16 (0.20)	−1.9 ((−4.6)–(−0.8))	0.168
Stride time variability (%)	10.38 (8.18)	10.80 (8.14)	−7.7 (−23.4–11.3)	0.401
Step regularity	0.68 (0.14)	0.64 (0.14)	5.8 (−1.3–13.4)	0.113
Stride regularity	0.70 (0.10)	0.67 (0.13)	5.0 (−0.2–10.4)	0.058
Step symmetry	0.97 (0.14)	0.96 (0.16)	0.8 (−3.6–5.3)	0.733
Stroop task				
Congruent stimuli				
1s	1.02 (0.27)	0.95 (0.32)	3.6 (−6.1–14.4)	0.478
2s	1.46 (0.52)	1.45 (0.47)	−2.2 (−11.5–8.0)	0.874
Variable	1.01 (0.25)	1.01 (0.25)	−0.8 (−10.7–10.1)	0.472
Incongruent stimuli				
1s	0.93 (0.27)	0.80 (0.27)	2.4 (−7.9–13.8)	0.656
2s	1.25 (0.45)	1.10 (0.41)	3.0 (−5.0–11.6)	0.664
Variable	0.80 (0.28)	0.74 (0.26)	−0.5 (−12.2–13.9)	0.937

*P* values in bold are significant differences between recurrent and non-recurrent fallers (*p* < 0.05). Data are presented as means (SD)

*1s* 1-s interval between stimuli, *2s* 2-s interval between stimuli, *Variable* variable interval between stimuli

### Effect of Stroop task on gait performance

**Fig. 1:**
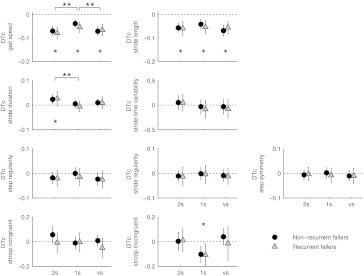
Dual-task costs in recurrent and non-recurrent fallers for gait and Stroop parameters. *Dotted line* depicts no dual-task costs (e.g., no difference between single and dual task). Positive dual-task costs indicate higher scores in dual-task condition compared to single-task condition. Data are log-transformed means and CI. *DTc* dual-task cost, *2s* 2 seconds interval between Stroop stimuli, *1s* 1 second interval between Stroop stimuli, *VS* variable intervals between Stroop stimuli. *Significant dual-task costs. **Significant differences between Stroop intervals

Dual-task costs are presented in Fig. 1. Adding the Stroop task to walking resulted in a significantly lower gait speed for all Stroop conditions (all *p* < 0.001). Stride length was significantly shortened during all Stroop conditions as well (all *p* < 0.001), but stride time was significantly shortened only in the 2-s Stroop condition (*p* = 0.006). Step regularity was negatively affected only in the variable Stroop interval condition (*p* = 0.027). Stride time variability, stride regularity, and step symmetry were not changed under dual task conditions in any of the Stroop conditions (all *p* ≥ 0.364).

The ANOVA-RM analysis yielded a main effect of Stroop condition on gait speed (*F*_2,259_ = 15.76, *p* < 0.001) and stride time (*F*_2,260_ = 7.216, *p* = 0.001), but not on all other gait parameters (all *p* > 0.008). Post hoc analyses revealed that dual-task costs for gait speed and stride time were higher in the 2-s interval compared to the 1-s interval condition (all *p* ≤ 0.001), and that dual-task costs for gait speed were higher in the variable interval than in the 1-s interval condition (*p* < 0.001).

### Effect of gait on Stroop task performance

Dual-task effects on Stroop task performance were only significant for the 1-s interval condition responding to incongruent stimuli (*t*_1,248_ = −3.700, *p* < 0.001, Fig. 1).

### Dual-task cost in recurrent fallers versus non-recurrent fallers

Dual-task effects on the different gait and Stroop parameters were compared between non-recurrent fallers and recurrent fallers using ANOVA-RM. This analysis yielded no significant group effects on gait speed (*F*_2,259_ = 0.20, *p* = 0.657), stride length (*F*_2,260_ = 0.02, *p* = 0.878), stride time (*F*_2,260_ = 0.05, *p* = 0.821), stride time variability (*F*_2,260_ = 0.23, *p* = 0.629), step regularity (*F*_2,260_ = 0.09, *p* = 0.768), stride regularity (*F*_2,260_ = 0.02, *p* = 0.876), or step symmetry (*F*_2,260_ = 0.014, *p* = 0.905). Likewise, dual-task costs for the Stroop task did not differ significantly between groups (*F*_2,260_ = 0.175, *p* = 0.676).

Because the recurrent fallers had higher UPDRS-III scores, slower TUG test performance, and lower MMSE scores, the analyses were repeated with these variables as covariates in the model. However, this did not alter our results in that no significant differences between recurrent fallers and non-recurrent fallers were found for any of the gait and Stroop outcomes.

### Descriptive analysis of priority

**Fig. 2:**
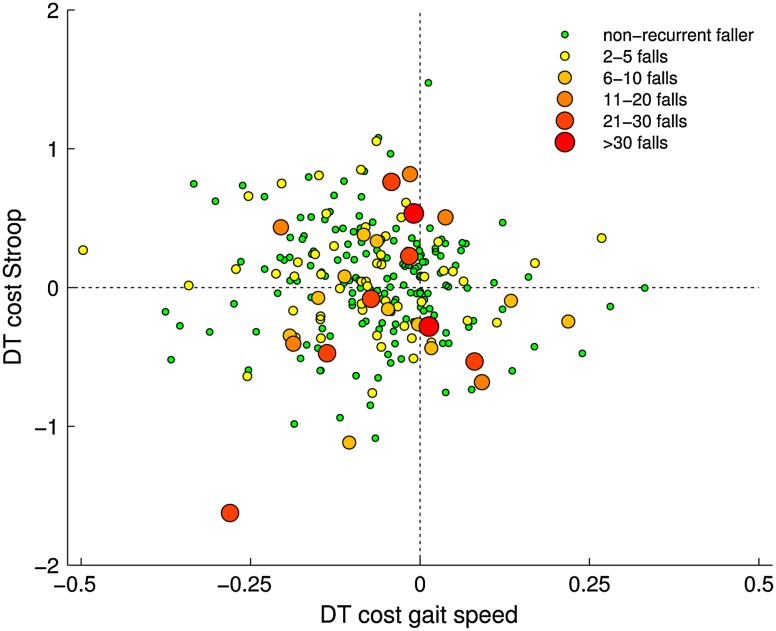
Dual-task costs for the Stroop task plotted against dual-task costs for gait speed for each individual. *DT* dual task

In order to analyze whether recurrent fallers used a different priority strategy under dual-task conditions compared to non-recurrent fallers, the individual dual-task costs for the Stroop task (2-s) were plotted against the dual-task costs for walking speed. As can be seen in Fig. 2, the positions of the recurrent fallers in the plot did not substantially differ from those of the non-recurrent fallers. Even in the group of frequent fallers (>5 falls/year; larger dots in Fig. 2), we could not determine different priority strategies (e.g., posture-second) compared to non-fallers.

## Discussion

In this large-scale study we evaluated whether dual-task performance was associated with future falls in patients with PD. The major finding was that patients with PD with recurrent falls did not have higher dual-task costs than patients without recurrent falls. This was found for all gait and Stroop outcomes. Second, recurrent fallers walked slower than non-recurrent fallers under single-task conditions and scored worse on clinical motor tests. Third, recurrent fallers did not use a different (e.g., posture-second) strategy in prioritizing the various tasks compared to non-recurrent fallers.

The similarity in dual-task costs between recurrent and non-recurrent fallers is largely in accordance with the only existing dual-task study to date that examined a small sample of fallers and non-fallers with PD [19]. This study reported similar dual-task effects on gait speed, stride length, stride time variability, and gait symmetry in both groups. This study, however, did find small, yet significant differences between fallers and non-fallers on swing time variability. We were unable to differentiate between swing and stance phase of the gait cycle and were therefore unable to replicate this finding.

In older people, significant associations between dual-tasking during walking and falls have been reported in a pooled analysis of different dual-task studies [4]. Importantly, only two studies have analyzed the added value of dual-task over single-task walking in predicting falls [32, 33]. In both studies, dual-task walking was as good in predicting fall risks as single-task walking. Another important observation was that dual-task walking only predicted falls in institutionalized elderly, as opposed to community-dwelling people. Thus, the predictive value of dual-task parameters for fall risk may be restricted to more frail elderly people than we studied in our present cohort of community ambulators.

Although recurrent fallers did not show different dual-task effects, they performed significantly worse on clinical motor tests and gait parameters than non-recurrent fallers. The most prominent differences between recurrent fallers and non-recurrent fallers were more severe motor symptoms (UPDRS-III), slower TUG performance, lower gait speed, and shorter stride length during single-task walking. These findings confirm those of previous studies demonstrating the predictive value of clinical balance and mobility measures [2, 34], and single-task walking for falls in PD [18, 19].

In addition to motor characteristics, cognitive dysfunction (and particularly executive dysfunction) predisposes patients with PD to falls [34, 35], perhaps because of difficulties in allocating and shifting attention in multiple-task situations [12]. It could therefore be expected that impaired executive function leads to difficulties in dual-task conditions and, consequently, may make participants more prone to falls. In our study sample of relatively early stage patients with PD, recurrent fallers showed lower performance on global cognition (MMSE), but differences in Stroop task performance were absent at baseline. Since the Stroop task relies on executive function, specifically reponse inhibition [36], the specific role of executive dysfunction in fall risk could not be confirmed in our study.

To gain insight into priority setting when allocating attention in multiple tasks, the dual-task costs for gait parameters were compared to those of the Stroop task. The “posture-second” hypothesis as suggested by Bloem implies that in dual-task conditions patients with PD do not adequately allocate attention to walking, placing them at risk of postural instability and falls [6]. Although we could not test this hypothesis statistically, the visualization of dual-task costs for both tasks in Fig. 2 does not provide support for this hypothesis. Patients in our cohort applied a variety of strategies, but recurrent fallers and non-recurrent fallers did not consistently show different preferences in the dual-task costs for gait compared to Stroop task performance. In order to further objectify priority strategy during multiple tasks, future research should focus on detecting reference values above which dual-task costs are detrimental for daily life gait and balance in healthy participants and people with gait and balance impairments.

Gait was slower under dual-task conditions presumably because of smaller stride lengths. This change in gait pattern implies that the attentional capacity was exceeded during dual tasking. Dual-task deficits in PD have been reported frequently in various combinations of tasks [37]. A neuroimaging study revealed that patients with PD showed increased brain activity while performing dual tasks compared to healthy participants [38], probably reflecting an attempt to compensate for dysfunction of the basal ganglia. Whether such dual-task abnormalities are caused by limited attentional resources, increased attentional demands for the separate tasks (due to less automatic movements), or from an impairment to switch between tasks remains to be clarified.

In contrast to our expectation, variability of gait was unaffected in the dual-task conditions. A cueing effect of the Stroop task may underlie this finding since an external cue can improve stride time variability in PD [28]. In order to detect a potential cueing effect induced by the Stroop task, we introduced a condition with variable intervals between stimuli. The mean interval of the variable-interval Stroop condition was comparable to the 2-s Stroop condition, and no differences between the two tasks were observed in the dual-task costs. However, this does not rule out the possibility of a cueing effect improving gait speed and variability in the faster 1-s Stroop task.

Some limitations of our study merit attention. Our cohort consisted of a large, homogeneous sample of mild to moderate patients with PD, all being community ambulators. Generalization to more severe patients with PD should, therefore, be done with caution. With disease progression, gait and postural deficits as well as cognitive impairments may result in larger dual-task costs that are potentially associated with falls. Also, all patients had to have a sedentary lifestyle in order to be eligible for the study. This selection may have influenced the incidence of falls, since an active lifestyle has been associated with reduced fall rates because of positive effects on strength and balance [39]. On the contrary, higher exposure to balance-threatening situations during exercise could increase the risk of falling. Importantly, even in this relatively ‘early’ and sedentary PD cohort, falls were common. Consequently, better identification of patients at risk to sustain a (first) fall is still needed in order to install fall prevention programs in a timely manner.

Another limitation of this study is that walking circumstances were fairly optimal. Participants walked over even ground without obstacles. In daily life, obstacles and uneven terrain have to be overcome while walking, leading to higher attentional demands. It is possible that dual-task deficits leading to instability and falls in daily life have remained undetected in this study because of the relatively simple walking task. Obstacle avoidance tasks or more challenging walking circuits are alternatives to be used in dual-task studies to further clarify the potential role of dual-task deficits in falling [11, 40]. Finally, we assessed gait variability as the average of two trajectories of 10 m, enabling us to measure this large sample of patients with PD. Ideally, a continuous walking distance of a minimum of 20 m is used to measure gait variability [41].

In conclusion, the present findings from this large cohort study do not support the use of dual-task paradigms for the prediction of falls in patients with mild to moderate Parkinson’s disease. With the current knowledge, future falls in community-dwelling patients with mild to moderate PD can be better predicted using relatively simple clinical tests such as the UPDRS and freezing of gait questionnaire [2].
